# G-Protein-Coupled Inwardly Rectifying Potassium (GIRK) Channel Activation by the p75 Neurotrophin Receptor Is Required for Amyloid β Toxicity

**DOI:** 10.3389/fnins.2017.00455

**Published:** 2017-08-08

**Authors:** Linda M. May, Victor Anggono, Helen M. Gooch, Se E. Jang, Dusan Matusica, Georg M. Kerbler, Frederic A. Meunier, Pankaj Sah, Elizabeth J. Coulson

**Affiliations:** ^1^Queensland Brain Institute, University of Queensland Brisbane, QLD, Australia; ^2^Clem Jones Centre for Ageing Dementia Research, University of Queensland Brisbane, QLD, Australia; ^3^Centre for Neuroscience, College of Medicine and Public Health, Flinders University Adelaide, SA, Australia; ^4^School of Biomedical Sciences, University of Queensland Brisbane, QLD, Australia

**Keywords:** p75^NTR^, amyloid β, Alzheimer's disease, neurodegeneration, excitotoxicity, potassium flux, Kir3, GIRK channel

## Abstract

Alzheimer's disease is characterized by cognitive decline, neuronal degeneration, and the accumulation of amyloid-beta (Aβ). Although, the neurotoxic Aβ peptide is widely believed to trigger neuronal dysfunction and degeneration in Alzheimer's disease, the mechanism by which this occurs is poorly defined. Here we describe a novel, Aβ-triggered apoptotic pathway in which Aβ treatment leads to the upregulation of G-protein activated inwardly rectifying potassium (GIRK/Kir3) channels, causing potassium efflux from neurons and Aβ-mediated apoptosis. Although, GIRK channel activity is required for Aβ-induced neuronal degeneration, we show that it is not sufficient, with coincident signaling by the p75 neurotrophin receptor (p75^NTR^) also required for potassium efflux and cell death. Our results identify a novel role for GIRK channels in mediating apoptosis, and provide a previously missing mechanistic link between the excitotoxicity of Aβ and its ability to trigger cell death pathways, such as that mediated by p75^NTR^. We propose that this death-signaling pathway contributes to the dysfunction of neurons in Alzheimer's disease and is responsible for their eventual degeneration.

## Introduction

Alzheimer's disease is a progressive neurodegenerative disorder that is characterized by deficits in memory and higher cognitive function. This cognitive decline is due to neurodegeneration, particularly of the hippocampus and entorhinal cortex, and loss of the cholinergic neurons of the basal forebrain. The brains of Alzheimer's disease patients contain deposits of aggregated amyloid β (Aβ), and a substantial body of work supports the hypothesis that Aβ underlies the etiology and pathogenesis of the disease (Hardy and Selkoe, 2002; Ballard et al., 2011). At the cellular level, the 42 amino acid form of Aβ (Aβ_42_) readily aggregates into soluble oligomers (Haass and Selkoe, 2007) which can directly contribute to neuronal and synaptic dysfunction and degeneration (Walsh et al., 2002; Haass and Selkoe, 2007; Walsh and Selkoe, 2007; Shankar et al., 2008). These Aβ-mediated changes, which include the loss of dendritic spines and neurites (Mucke et al., 2000; Smith et al., 2009; Perez-Cruz et al., 2011), have been proposed to cause the cognitive decline, which precedes neuronal death in Alzheimer's disease (Ondrejcak et al., 2010; Palop and Mucke, 2010).

Neuronal degeneration in the context of Aβ is linked to the excitotoxic activities of the peptide, whereby an increase in intracellular calcium induced by Aβ triggers apoptosis by an ill-defined pathway (Palop et al., 2007). An alternative mechanism of Aβ-induced neurodegeneration is via the p75 neurotrophin receptor (p75^NTR^). p75^NTR^ initiates apoptosis in a variety of neurodegenerative conditions, including apoptotic death triggered by oligomeric forms of Aβ_42_ *in vitro* (Yang et al., 2008; Coulson et al., 2009) and in animal models of Alzheimer's disease (Sotthibundhu et al., 2008; Knowles et al., 2009; Wang et al., 2011). p75^NTR^ is expressed throughout life in basal forebrain cholinergic neurons and is also ectopically upregulated in response to Aβ accumulation in other disease-vulnerable brain areas, such as the cortex and hippocampus (Mufson and Kordower, 1992; Chakravarthy et al., 2010; Perez et al., 2011), and in other excitotoxic disease conditions such as motor neuron disease and epilepsy (Ibanez and Simi, 2012).

Although, the mechanism by which p75^NTR^ mediates cell death triggered by Aβ is unclear (Costantini et al., 2005; Coulson, 2006), we have previously demonstrated that neurotrophin signaling through p75^NTR^ can activate G-protein activated inwardly rectifying potassium (GIRK) channels, triggering a cell death pathway (Coulson et al., 2008). GIRK channels are typically activated by inhibitory neurotransmission, allowing transitory potassium efflux, which lowers the neuronal resting membrane potential and dampens neuronal excitability (Dascal, 1997). Activation of GIRK channels is required for some forms of synaptic plasticity such as long-term potentiation (LTP) (Chung et al., 2009a), and GIRK expression can be regulated by enhanced glutamatergic activation (Chung et al., 2009b). As Aβ is known to cause excitotoxicity at least partially via glutamatergic processes (De Felice et al., 2007; Alberdi et al., 2010), these converging lines of evidence suggest that activation of the p75^NTR^-GIRK pathway by excitotoxic Aβ_42_ may play a direct role in neuronal degeneration. We therefore investigated whether Aβ treatment of neurons affects GIRK channel expression and activity, and whether the resultant changes lead to neuronal degeneration.

## Materials and methods

### Primary culture

All animal procedures were approved by the University of Queensland Animal Ethics Committee. Pregnant C57Bl6 mice were sacrificed and their E18 embryos were removed by Caesarian section. Hippocampal tissue was dissected from each embryonic brain, chopped, and digested with 0.05% trypsin (Gibco). Neurons were then dissociated by serial trituration, passed through a 40 μm filter and resuspended in medium. Neurons were plated on poly-L-lysine-coated tissue culture dishes, coverslips, or 3 cm MatTek glass-bottom dishes (MatTek Corp) and cultured in medium containing Dulbecco's modified Eagle's medium (DMEM)/Ham's F12 (Gibco), 10% fetal bovine serum (FBS; JRH Biosciences) and 2 ng/ml brain-derived neurotrophic factor (BDNF). This medium was then changed the next day to neurobasal medium (Gibco) supplemented with B27 (Gibco) and 2 ng/ml BDNF, and the cells were cultured for 10–24 days at 37°C and 5% CO_2_ with medium changes as required. For short-term, lower density cultures, neurons were plated in DMEM/Ham's F12 medium containing 10% NeuroCult (StemCell Technologies) and 2 ng/ml BDNF (Millipore) in tissue culture dishes coated with 0.1 mg/ml poly-L-lysine (Sigma).

### Treatments

Aβ peptides were synthesized using t-Boc chemistry and purified using reverse phase HPLC by Dr. James I. Elliott at Yale University. To prepare Aβ peptides for treatment, both Aβ_42_ and Aβ_16_ were reconstituted in sterile water to 200 μM stock solution, incubated overnight at 4°C and used at 20 μM final concentration in medium or electrophysiological bath solution. Fresh solutions were made for each assay. Co-treatments were added once to neuronal cultures at *t* = 0, the time of Aβ treatment, unless otherwise stated. APV (30 μM; Sigma) was used to block N-methyl-D-aspartate (NMDA) receptors. TertiapinQ (100 nM; Alomone) was used to block GIRK channel activity. To activate GABA_B_ receptors and down-regulate GIRK channels, baclofen (Sigma) was used at 50 μM. The antagonist CGP55845 (1 μM; Tocris) was used to block GABA_B_ receptor activity. The metalloprotease inhibitor TAPI-2 (20 μM; Calbiochem) was used to prevent cleavage of p75^NTR^; the initial treatment was done at *t* = 0 and a second treatment of TAPI-2 was performed 6 h later. The c29 peptide used to block p75^NTR^ death signaling was a 29 amino acid residue peptide of the juxtamembrane Chopper domain (Coulson et al., 2000; Matusica et al., 2013; KRWNSCKQNK QGANSRPVNQ TPPPEGEKL) fused to a non-naturally occurring protein transduction domain peptide (YARAAARNARA) based on PTD4 (Ho et al., 2001). The control used for c29 was a scrambled version of the peptide (SKGQVCRNQP GQNKPEPANK SWKETPLRN) fused to the transduction domain sequence and a fluorescent indicator (FITC; Matusica et al., 2013). These peptides were also synthesized at Yale University.

### Aβ SDS-PAGE and western blotting

To identify the aggregation state of Aβ used in these assays, Aβ peptides were separated by SDS-PAGE and detected by western blotting. HPLC-purified Aβ solution was prepared as described above and samples were mixed with LDS sample loading buffer and loaded onto Nupage precast BisTris gels. SDS-PAGE was conducted in Nupage MES or MOPS SDS running buffer, after which proteins were transferred to an Immobilon® membrane (BioRad) in 20% methanol. Blots were incubated with primary Aβ antibody (1:500, clone 6F/3D to residues 8–17; Dako) followed by horseradish peroxidase-conjugated secondary antibodies in phosphate-buffered saline (PBS) containing 0.05% Tween 20, after which Aβ-immunoreactive bands were visualized with SuperSignal® West Pico or Femto Chemiluminescent Substrate (Thermoscientific), according to the manufacturer's instructions. Blots were exposed to film and developed by X-ray developer.

### Calcium imaging

To measure calcium flux, cultured neurons were loaded with the calcium indicator Oregon Green 488® BAPTA-1 AM (5 μM; Invitrogen) for 1 h prior to imaging. They were then washed 3 times with medium and imaged on a Marianas TIRF/FRET/FRAP inverted high-speed imaging fluorescence microscope at 37°C and 5% CO_2_. Neurons were imaged for ~2 min to obtain baseline calcium fluorescence, then treated with Aβ peptides and the NMDA receptor antagonist APV as indicated, before being imaged 5 min later for ~2 min (for APV) and/or 15 min later (for Aβ). Calcium flux was observed in both short-term and long-term cultures. Although, the short-term cultures were spontaneously active, the synchronous firing of the long-term (21–24 days) cultures facilitated quantification of calcium flux, as a result of which long-term cultures were used for these experiments. Fluorescence values of all cells in the field of view (at least three fields of view per condition) of mature cultures were quantified using Slidebook software, and the data were pooled within each condition and analyzed using one-way analysis of variance (ANOVA).

### Surface biotinylation assay

The levels of surface GIRK1 and GIRK2 expression were determined by surface biotinylation assay as described previously (Anggono et al., 2011). Briefly, mouse hippocampal neurons cultured for 12–15 days were treated with Aβ_16_ (20 μM), Aβ_42_ (20 μM), or Aβ_42_ (20 μM) plus baclofen (50 μM) for 2 h. Neurons were subsequently washed twice with artificial cerebrospinal fluid (ACSF) containing (in mM), 25 HEPES, 120 NaCl, 5 KCl, 2 CaCl_2_, 2 MgCl_2_, and 30 D-glucose, pH 7.4, and incubated with 1 mg/ml Sulfo-NHS-SS-Biotin (Pierce) for 30 min on ice. Free biotin was quenched by washing cells twice with ice-cold Tris-buffered saline (pH 7.4). Cultures were lyzed and sonicated in RIPA buffer and incubated with Neutravidin beads (Pierce) for 3 h at 4°C. Beads were washed three times and eluted with 2 X SDS sample buffer, followed by western blotting analyses using rabbit polyclonal antibodies against GIRK1 (Alomone, APC-005, 1:1,000 or Abcam, ab129182, 1:2,000) and GIRK2 (Alomone, APC-006, 1:500) followed by anti-rabbit HRP-linked secondary antibodies (1:10,000) from GE Healthcare. Bands were visualized using the ECL detection method and X-ray film. For quantification, total and surface GIRK1/2 levels were normalized to actin, and the data are presented as % change relative to Aβ_16_.

### Electrophysiology

C57Bl6 mice (3–4 weeks old) were deeply anesthetized using isoflurane inhalation and decapitated. The brain was then quickly removed and submerged in ice-cold, oxygenated ACSF containing (in mM): 87 NaCl, 25 NaHCO_3_, 25 glucose, 50 sucrose, 2.5 KCl, 1.2 NaH_2_PO_4_, 4 MgCl_2_, 0.5 CaCl_2_, pH 7.4. Coronal brain slices (300 μm) containing the CA1 region of the rostral hippocampus were prepared using a vibratome (VT1000S, Leica) and transferred for incubation at 36°C for 30 min in oxygenated ACSF containing (in mM): 118 NaCl, 25 NaHCO_3_, 10 glucose, 2.5 KCl, 2.5 CaCl_2_, 1.2 NaH_2_PO_4_ and 1.3 MgCl_2_, pH 7.4 (95% O_2_/5% CO_2_). Slices were then equilibrated to room temperature for at least 30 min, before being transferred to the immersion recording chamber. During recordings, slices were continuously perfused with oxygenated ACSF (95% O_2_/5% CO_2_) at 34°C and secured with a platinum harp strung with parallel nylon threads. The CA1 region was visualized under low magnification using an upright microscope (5x magnification; BX50WI, Olympus) and pyramidal neurons of the CA1 *stratum pyramidale* layer were observed at high magnification (40x) using differential interference contrast (DIC) optics combined with infrared illumination. Somatic whole-cell recordings were performed using 3–5 MΩ borosilicale pipettes filled with potassium-based internal solution containing (in mM): 135 KMeSO_4_, 8 NaCl, 10 HEPES, 2 Mg_2_-ATP, 0.3 Na_3_-GTP, 0.1 spermine, 7 phosphocreatine, and 0.3 EGTA (pH 7.3 with KOH, osmolarity 280–290 mOsm). GABA_B_-receptor mediated slow inhibitory postsynaptic currents (sIPSCs) were recorded from pyramidal neurons in voltage-clamp at a holding potential of −50 mV, and were evoked by the placement of a concentric bipolar stimulating electrode (World Precision Instruments) in the *stratum radiatum* to stimulate Schaffer collaterals and local γ-aminobutyric acid (GABA)-expressing interneurons with single pulses or train stimulation (20 Hz; Scanziani, 2000). Holding currents were monitored and recorded in voltage-clamp at a holding potential of −60 mV. Aβ_42_ (20 μM), the GABA_B_-selective antagonist CGP55845 (1 μM), baclofen (50 μM), tertiapin (100 nM), 2-amino-5-phosphonopentanoic acid (APV; 50μM), and ZD7288 (4-ethylphenylamino-1,2-dimethyl-6-methylaminopyrimidinium chloride, 10 μM) were bath applied where indicated. During GABA_B_ recordings, picrotoxin (50 μM) was added to the perfusing ACSF solution to block ionotropic GABA_A_ receptors. Signals were amplified using a Multiclamp 700B amplifier (Molecular Devices), current signals were filtered at 4–8 kHz, digitized at 20 kHz using an Instrutech ITC-18 interface, and acquired on an iMac using Axograph X. Access resistance (4–18 MΩ, uncompensated) was monitored throughout the experiment. Modulation of the GIRK current reversal potential by high external potassium was performed using primary hippocampal cultures, prepared as described above, and the osmolarity of high KCl (20 mM) ACSF was balanced by an equivalent reduction in NaCl molarity.

### Potassium imaging

To quantify change in the intracellular potassium concentration, neurons that had been cultured for 3 days were incubated with 2 μM Asante Potassium Green-2 (Teflabs) indicator for 30 min. After exchange of the medium, cultures were left to recover for a minimum of 15 min in an incubator before being transferred to the Axio observer microscope chamber held at 37°C and 5% CO_2_. Fluorescence (488 nm) and DIC (Nomarski) images were captured every 5 min for 3 h. Test compounds, including Aβ_42_, were added 15 min after imaging had commenced. The fluorescence values of all cells in the field of view (six fields of view per condition) were quantified using Imaris software from images taken 5 min before the addition of the test compounds (time 1) and 30, 110, or 160 min after treatment (time 2). The percentage change in fluorescence over time for each cell was then calculated.

### *In vitro* apoptosome assay

Apoptosome components were isolated from the soluble fraction of cell lysates obtained from nerve growth factor (NGF)-differentiated PC12 cells. PC12 cells were maintained at 37°C with 10% CO_2_ in DMEM supplemented with 10% horse serum (Sigma) and 5% FBS before being differentiated with 50 ng/ml NGF in DMEM supplemented with 0.1% horse serum for 2 days. They were then collected by scraping, washed with ice cold PBS, and centrifuged to form tight cell pellets which were weighed and resuspended 1:1 with hypotonic extraction buffer containing (in mM): 5 EGTA, 50 PIPES, 2 MgCl_2_, 1 DTT, 0.1 PMSF, pH 7. Cells were allowed to swell on ice for 25 min before being sheared with 100 strokes of a B-type pestle in a Dounce homogenizer (Kimble-Kontes). The solution was centrifuged at 100,000 g for 1 h at 4°C, after which the supernatant was aliquoted and stored immediately at –80°C. Lysate containing 100 μg protein was incubated in a 50 μl reaction mix with cytochrome C (0, 0.5, 1.0, 1.5, or 2 μM), 0.5 μM dATPs, and the fluorescent caspase substrate Ac-DEVD-AMC (100 μM), with KCl added to a concentration of 20, 50, 80, 110, or 140 mM. All conditions were assessed in triplicate. Cleavage of the substrate was measured by a Polarstar Optima plate reader every 5 min for 1 h (excitation filter, 380 ± 10 nm; emission filter, 460 nm); triplicate readings were pooled for each condition.

### Neurite outgrowth assay

Neurons were plated at a density of 40,000 cells per well of a 24-well plate (Falcon) and grown for ~3 weeks. They were then treated with Aβ, baclofen and/or CGP55845. After 20 h, neurons were washed, fixed with 4% paraformaldehyde, immunostained for β-III tubulin and co-stained with DAPI. Three random fields of view were imaged for each condition per experiment; images were overlaid with a grid and the neurite crossings were counted and divided by the number of neurons per field (DAPI- and β-III tubulin-positive soma) to give a ratio of neurites per neuron. Ratios were averaged across conditions and data were analyzed by one-way ANOVA.

### *In vitro* neuronal survival assays

To determine the level of neuronal survival, low density hippocampal cultures were plated at 40,000 cells per 11 mm diameter well of a 4-well plate (Cell-star®, Greiner) with a grid marked for later cell identification. Cells were cultured overnight in growth medium at 37°C and 5% CO_2_. The number of neurons within a fixed grid quadrant was counted 24 h after plating (*t* = 0) and the same quadrant was counted again 20 h later (*t* = 20). Live cells were determined by morphology, and, in some experiments, also by exclusion of propidium iodide. Similar results were obtained when MTT [3-(4,5-dimethylthiazol-2-yl)-2,5-diphenyltetrazolium bromide] was used to identify live cells in the cultures. All treatments were applied immediately after the initial cell count and remained in the culture medium until the end of the experiment. Survival was expressed as the percentage of neurons remaining alive at *t* = 20. Under each condition a minimum of 250 neurons to a maximum of 600 neurons were counted across 4 different gridded wells at *t* = 0. Each condition was replicated a minimum of 3 times (*N* = 3), with neurons derived from different litters on different days.

### p75^NTR^ cleavage experiments

p75^NTR^- and TrkB-expressing NSC-34 cells were grown to 60% confluence in T25 flasks at a density of 5 × 10^7^ cells/flask in DMEM supplemented with 10% FBS and 1% penicillin/streptomycin/glutamine solution (PSG) at 37°C and 5% CO_2_, on a poly-D-lysine (100 μg/ml) and rat-tail collagen (50 μg/ml) matrix, supplemented with 2 μg of laminin. Cells were differentiated by exchange of medium to 1:1 DMEM:Ham's F12, plus 1% N-2 supplement, 1% PSG, 1% modified Eagle's medium non-essential amino acids, and 1 μM retinoic acid for 3 days. Cells were then treated with 20 μM Aβ_42_ or 20 μM Aβ_16_ either alone or in combination with the TAPI compound for 3 h. To inhibit degradation of the p75^NTR^ intracellular domain fragment cells were treated with 5 μM clasto-lactacystin β-lactone (Calbiochem) and 1 μM epoxomicin (Sigma) 1 h prior to addition of other compounds. Cells were treated with 200 nM of phorbol 12-myristate 13-acetate (PMA; Sigma) as a positive control for p75^NTR^ cleavage. They were then lyzed using chilled lysis buffer containing 10 mM Tris-HCl, pH 8.0, 150 mM NaCl, 2 mM EDTA, 1% NP-40, 1% Triton X-100, 10% glycerol, 1 mM phenylmethanesulfonyl fluoride, 1 mM sodium orthovanadate, 1 μM batimastat (BB-94), and 1% Roche protease inhibitor cocktail. Following electrophoresis of cell lysates through 4–12% Bis-Tris buffered SDS gels (Life Sciences), the following antibodies were used for western blotting: rabbit anti-p75^NTR^ intracellular domain #9992 (1:5,000; a kind gift from Prof Moses Chao, Skirball Institute), mouse anti-β-III tubulin (1:1,000; Promega), and LICOR donkey anti-rabbit 680 or donkey anti-mouse 800 secondary antibody (1:50,000; Invitrogen). Blots were visualized with Supersignal West Femto Sensitivity Substrate (Pierce).

### Statistical analysis

Statistical analyses were performed using Prism 4 for Macintosh (GraphPad Software, Inc.). Two group comparisons were made using *t*-tests. For multiple comparisons, data were analyzed by ANOVA conducted using Newman–Keuls post-test comparisons, except for the potassium imaging data which were analyzed using Tukey's multiple comparisons post-test. All graphs are mean ± SEM.

## Results

### Aβ upregulates GIRK channel surface expression and activity

**Figure 1:**
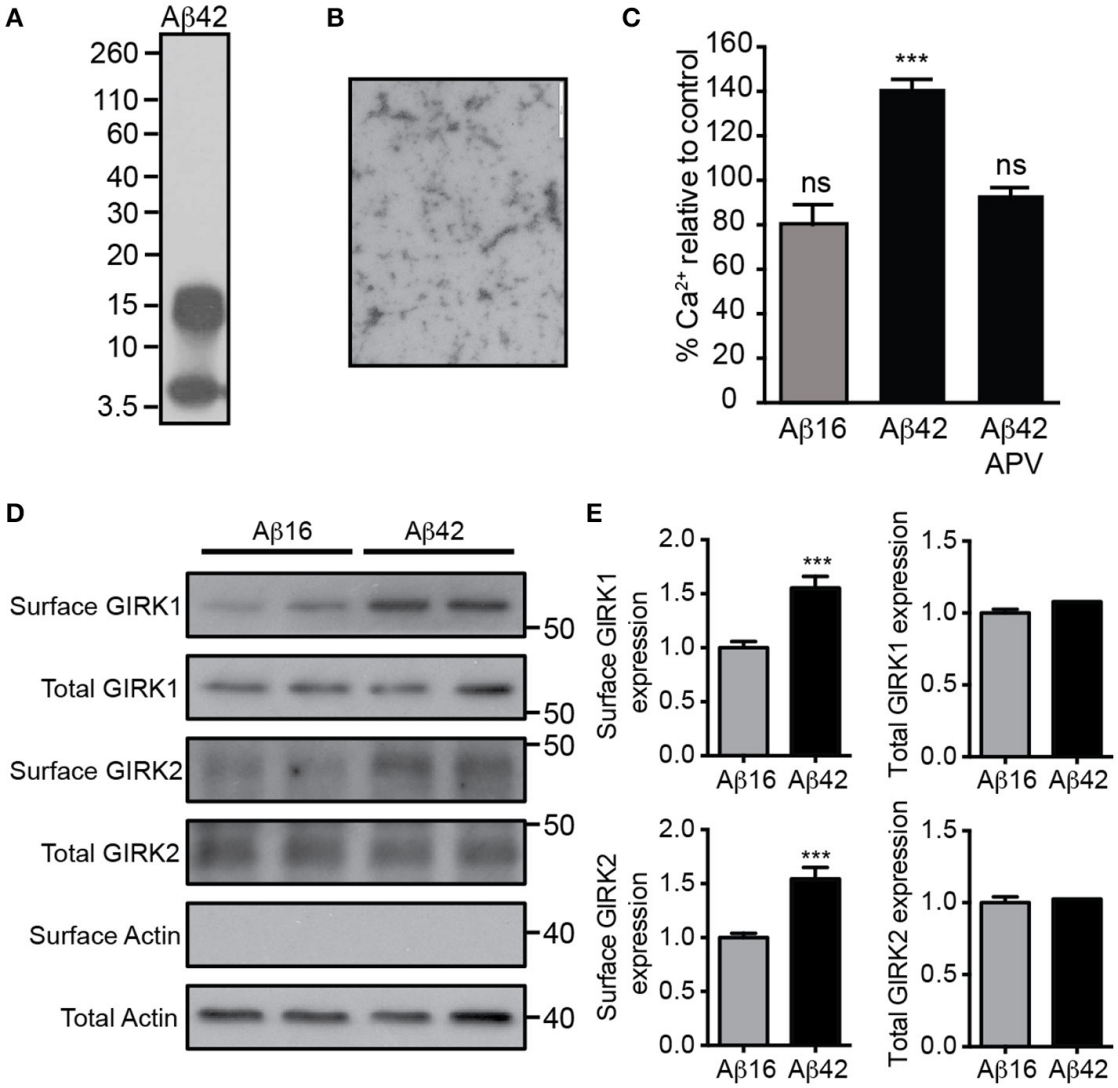
Excitotoxic oligomeric Aβ_42_upregulates GIRK channel surface expression. **(A)** Western blot of the preparation of purified Aβ_42_ peptide used in this study demonstrating that it is predominantly in an oligomeric (15 kDa) or monomeric (4 kDa) form. **(B)** A transmission electron photomicrograph of the Aβ_42_ peptide preparation. **(C)** Quantification of relative [Ca^2+^]_i_ of spontaneously active cultured neurons following treatment with Aβ_16_, Aβ_42_, or Aβ_42_ and the NMDA receptor antagonist APV as indicated. The fluorescence intensity of the Oregon Green 488 BAPTA -AM almost doubled in neurons treated with Aβ_42_ in comparison to the untreated baseline (*n* = 59 neurons). Pre-treatment with the NMDA receptor inhibitor APV prevented the Aβ_42_-induced calcium increase (*n* = 69 neurons, ^***^p < 0.001), with calcium levels being similar to those of controls. Western blot **(D)** and quantification **(E)** of total and surface GIRK1 and GIRK2 protein levels in hippocampal neurons treated for 2 h with either control Aβ_16_ or oligomeric Aβ_42_ (*n* = 12 replicates; ^***^p < 0.001; Mann–Whitney *U*-test).

We first prepared a solution of Aβ_42_ that contained predominantly low-order oligomers and monomers (Figures 1A,B). Treatment of cultured embryonic mouse hippocampal neurons with 20 μM Aβ_42_, but not the control Aβ_16_ peptide, raised the neuronal intracellular calcium concentration (Figure 1C), indicating that it had excitotoxic properties. Furthermore, the calcium increase induced by Aβ_42_ occurred most obvious in spontaneously active neuronal cultures (data not shown) and was inhibited by the NMDA receptor antagonist APV (Figure 1C), indicating glutamate receptor activity was required for the excitotoxicity. As it has been shown that over-activation of the NMDA receptor increases the number of GIRK channels at the surface of neurons (Chung et al., 2009b), we investigated the effect of Aβ_42_ on GIRK channel expression in cultured embryonic mouse hippocampal neurons. Western blot analysis demonstrated that surface GIRK1 and 2 protein levels were significantly increased in neurons 2 h after Aβ_42_, but not control Aβ_16_, application (Figures 1D,E). Total cellular levels of GIRK1 and 2 subunits were unchanged by the treatments (Figures 1D,E). These data indicate that Aβ_42_ causes a rapid redistribution of existing GIRK subunits into the plasma membrane.

**Figure 2:**
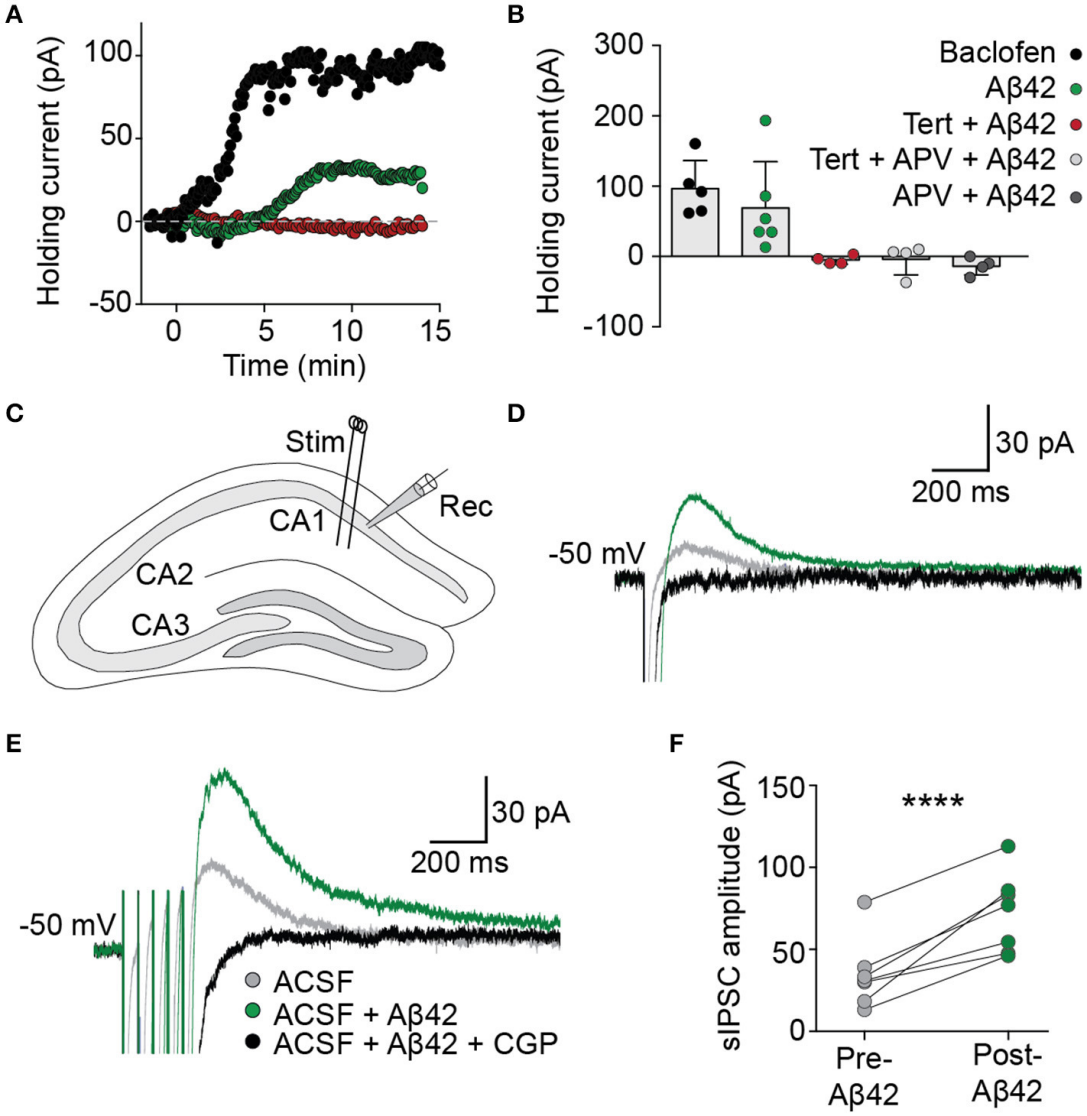
Aβ_42_ upregulates GIRK channel activity. **(A)** Bath application of Aβ_42_ (20 μM) at time = 0 evoked an outward current in CA1 pyramidal cells (green trace), analogous with the GABA_B_ receptor agonist baclofen (50 μM; black trace), when recorded in voltage-clamp at holding potential −60 mV. Aβ_42−_induced efflux was not observed in cells previously incubated in the specific GIRK channel antagonist tertiapin (100 nM; red trace). **(B)** Summary plot to show changes in averaged holding current from single cells 15 min after the application of baclofen (97 ± 18 pA, *n* = 5; black), Aβ_42_ (69 ± 27 pA, *n* = 6/7 cells; green), tertiapin-incubated Aβ_42_ application (−5 ± 3 pA, *n* = 4 cells; red), tertiapin- and APV-incubated Aβ_42_ application (−5 ± 11 pA, *n* = 4 cells; light gray) and APV-incubated Aβ_42_ application (−14 ± 6 pA, *n* = 4; dark gray). **(C)** Schematic representation indicating electrode placements used to evoke (Stim) and record (Rec) slow inhibitory postsynaptic currents (sIPSCs) within coronally sectioned hippocampal tissue. Representative single-pulse **(D)** and train-stimulated (20 Hz; **E**) sIPSC traces before (gray) and after (green) application of bath applied Aβ_42_(20 μM), blocked by GABA_B_ receptor antagonist CGP55845 (1 μM; black), recorded at a holding potential of −50 mV. **(F)** Summary plot of averaged sIPSC peak amplitude responses for all tested CA1 pyramidal neurons, before and after bath application of Aβ_42_ (one-way ANOVA, Bonferroni test, ^****^p < 0.0001, *n* = 7).

The functional effect on basal GIRK channel activity of the Aβ_42_-induced increase in membrane GIRK subunits was examined in acute brain slices using whole-cell voltage-clamp recordings from CA1 pyramidal neurons. Bath application of Aβ_42_ led to a slowly developing outward current when the cells were voltage-clamped at resting membrane potentials (69 ± 27 pA, *n* = 6/7; Figures 2A,B), analogous to that induced by an acute treatment with baclofen, a GABA_B_ receptor agonist that activates GIRK channels (97 ± 18 pA, *n* = 5). This Aβ_42_-induced outward current was induced within 5 min of application, indicating that GIRK channel upregulation occurred relatively quickly in response to Aβ_42_ treatment. Following Aβ_42_ treatment, a number of neurons became hyperpolarized, indicating efflux of potassium (data not shown). The effect of Aβ_42_ on holding currents was blocked by prior incubation in the presence of the potassium channel blocker barium (BaCl_2_ + Aβ_42_, −7.4 ± 4.9 pA, *n* = 2; BaCl_2_, −50.5 ± 8.8 pA, *n* = 5). It was also blocked by tertiapin (>30 min incubation, −5 ± 3 pA, *n* = 4), the NMDA receptor antagonist APV (−14 ± 6 pA, *n* = 4) or tertiapin plus APV combined (−4 ± 11 pA, *n* = 4; Figures 2A,B).

We next investigated the effect of acute Aβ_42_-induced upregulation of GIRK channels on hippocampal circuit activity via synaptically evoked GABA_B_ GIRK currents within the hippocmapus. In the presence of picrotoxin to block GABA_A_ receptor-mediated synaptic currents, electrical stimulation of the Schaffer collaterals generated slow IPSCs in CA1 hippocampal neurons when voltage-clamped at a holding potential of −50 mV (Figure 2C). Bath application of Aβ_42_ significantly increased the amplitude of sIPSCs (green trace; one-way ANOVA, Bonferroni test, *p* < 0.0001, *n* = 7; Figures 2D–F), which were completely blocked by bath application of the GABA_B_-selective antagonist CGP55845 (1 μM; Figures 2D–F). This effect on the synaptically evoked GIRK currents was observed from as early as 8 min after Aβ_42_ application and was sustained for the duration of the recording (up to 40 min). These data indicate that synaptically activated GIRK channel currents are upregulated following Aβ_42_ treatment, leading to enhanced slow inhibitory neurotransmission in the CAI hippocampal circuit.

### GIRK channel activity results in lowered intracellular potassium

**Figure 3:**
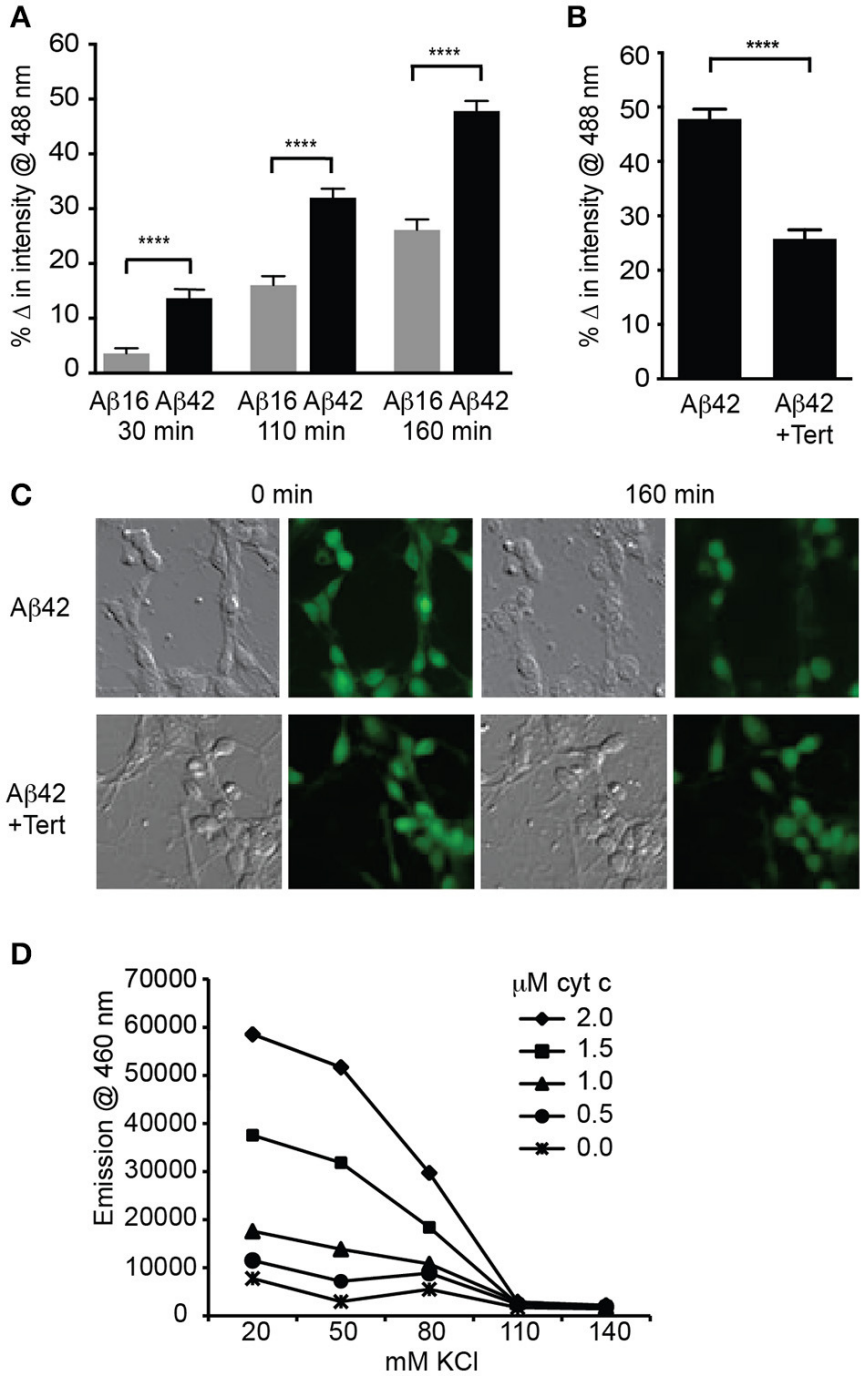
GIRK channel activity results in lowered intracellular potassium, a prerequisite for apoptosome activity. **(A)** Average decrease in potassium concentration within individual cultured hippocampal neurons, as measured by the fluorescence of the potassium indicator Asante Potassium Green-2 at each of the indicated times after Aβ application (*n* = 502 cells; ^****^p < 0.0001). **(B)** Average potassium concentration decrease in cultured neurons treated with Aβ_42_ and the GIRK channel inhibitor tertiapin (Tert) for 160 min (*n* = 538 neurons; ^****^p < 0.0001). **(C)** Representative relief contrast and fluorescence (Asante Potassium Green-2) photographs of neuronal cultures taken immediately and 160 min after Aβ and tertiapin treatment. **(D)** Graph of caspase activity, detected by cleavage of the fluorogenic substrate Ac-DEVD-AMC (emission at 460 nm), in lysates of NGF-differentiated PC12 cells added to different concentrations of KCl buffers (K^+^ concentration ranging from 20 to 140 mM), and incubated in the presence of a range of cytochrome c concentrations (0–2 μM). Neuronal physiological K^+^ concentrations (>110 mM) completely suppressed caspase activity regardless of cytochrome c concentration, and maximal caspase activity occurred in the lowest (20 mM) K^+^ concentration. Cytochrome c concentrations higher than 1 μM combined with K^+^ concentrations lower than 80 mM allowed caspase activity to occur (data points are the average of triplicates; all non-overlapping data points at these concentrations are significantly different at *p* ≤ 0.01).

We next asked if significant potassium efflux occurred via potassium channels following Aβ treatment by measuring the intracellular potassium concentration using a fluorescent indicator APG-2. APG-2 fluorescence declined over time in all conditions over the course of 160 min, likely due to potassium efflux through leak channels (Figure 3A). However, the intracellular fluorescence of Aβ_42_-treated neurons significantly decreased compared to that of Aβ_16_-treated neurons (Figure 3A), indicating a considerable loss of intracellular potassium in the former condition. As it has been determined that the APG-2 fluorescence is saturated at potassium concentrations higher than ~80 mM (Rimmele and Chatton, 2014), this result indicates that the internal potassium concentration of many of the Aβ_42_-treated neurons was falling below 80 mM. To determine whether the potassium efflux might be mediated via GIRK channels, neurons were treated with the most specific GIRK channel inhibitor, tertiapin, which significantly inhibited the Aβ_42_-induced reduction in intracellular potassium (Figures 3B,C).

Activation of caspases by the apoptosome can be regulated by potassium levels (Yu and Choi, 2000; Cain et al., 2001; Coulson et al., 2008). To examine whether the reduced level of intracellular potassium in the neurons treated with Aβ was capable of mediating caspase activation, we determined the relationship between potassium concentration and apoptosome formation. This was achieved by using a cell-free assay in which the core mitochondrial cell death machinery of the apoptosome, comprising cytochrome c, caspase 9, and Apaf1 (Riedl and Salvesen, 2007), was derived from cell lysates. The ability of these components to form an apoptosome and cleave a fluorogenic caspase 3 substrate was measured in decreasing concentrations of potassium buffer and increasing concentrations of exogenous cytochrome c. Physiological levels of potassium (110–140 mM; Yu and Choi, 2000) were able to prevent all apoptosome activity regardless of the cytochrome c concentration (Figure 3D). By contrast, 80 mM potassium or less resulted in significant caspase activity, indicating permissiveness for apoptosome formation (Figure 3D). These *in vitro* assays demonstrated that a potassium concentration approaching half that typically found in healthy neurons, and equivalent to that induced in Aβ_42_-treated neurons, is necessary for activation of the apoptosome by cytochrome c, which in turn is a prerequisite for apoptosis via the mitochondrial cell death pathway (Cain et al., 2001).

### GIRK channel activity is required for Aβ_42_-induced neuronal degeneration

**Figure 4:**
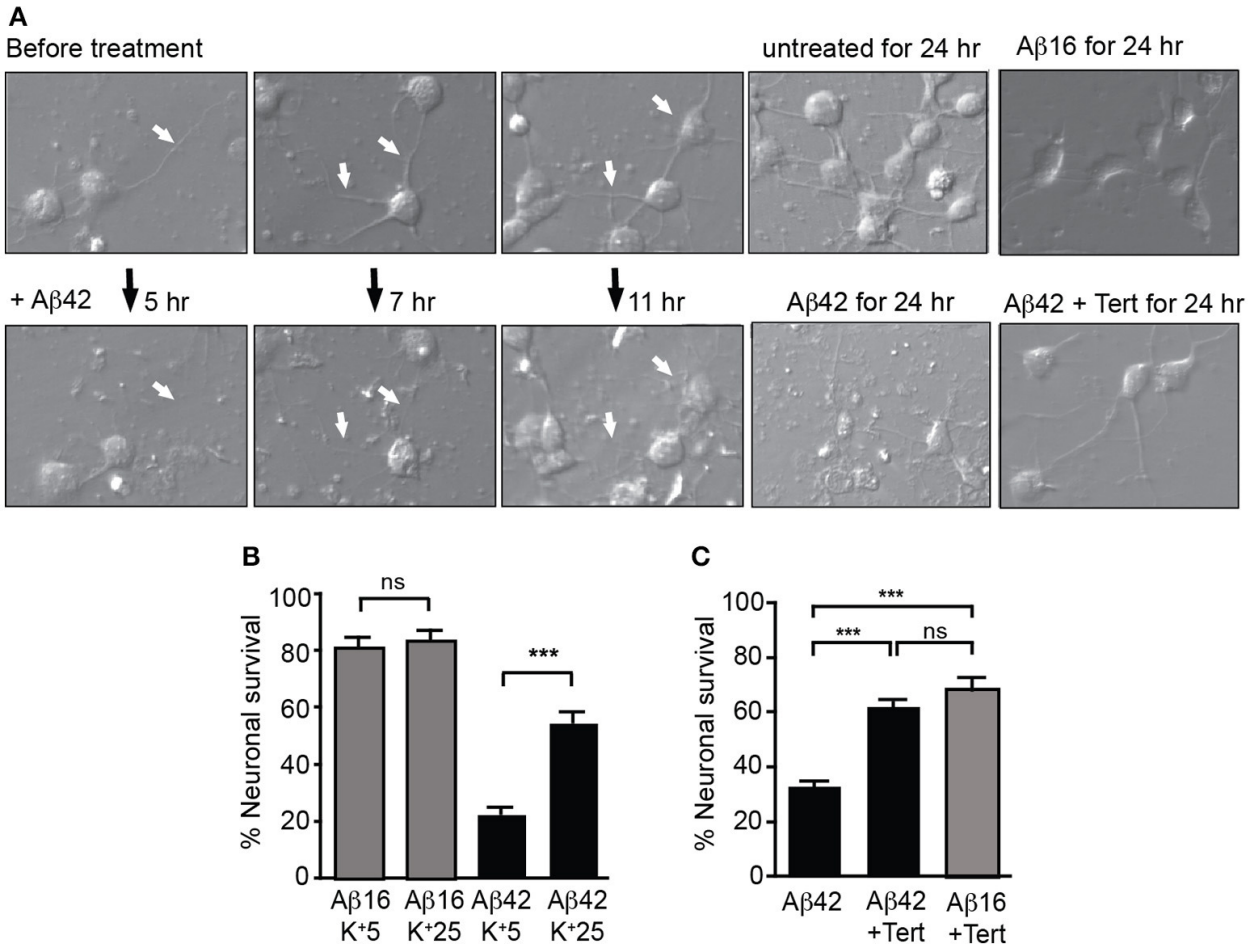
GIRK channel activity is required for Aβ_42_-induced neuronal death. **(A)** Images of neuronal degeneration in hippocampal cultures that were treated with Aβ_42_ or Aβ_16_ 24 h after culture establishment. Neurites (highlighted by white arrows) showed considerable degeneration within 5 h of Aβ_42_ application with obvious neurite loss and some blebbing as early as 7 h after Aβ_42_ treatment. Neurons that had established extensive neuritic networks prior to treatment also showed neuritic loss and blebbing 11 h after Aβ_42_ application. Neurons cultured for 24 h without Aβ (untreated) or in the presence of Aβ_16_ developed healthy neuritic networks. Treatment of cultured neurons with Aβ_42_ for 24 h resulted in neuronal loss, with most surviving neurons displaying apoptotic morphology. In contrast, fewer cells displayed degenerative morphological changes in Aβ_42_-treated cultures with co-application of tertiapin (Tert) **(B)** Percentage survival of neurons cultured in the presence of Aβ in either typical potassium concentrations (5 mM; K^+^5), or high potassium (25 mM; K^+^25) for 20 h; the latter condition inhibited intracellular potassium efflux from cells. Aβ_42_-initiated neuronal death was inhibited in the presence of 25 mM potassium (*N* = 3 experiments). **(C)** Percentage survival of neurons cultured in the presence of Aβ and tertiapin (Tert) for 20 h. Aβ_42_-initiated neuronal death was inhibited when GIRK channels were blocked with tertiapin (*N* = 6 experiments). ^***^p < 0.001, *ns*, not significant.

We next treated neuronal cultures with Aβ overnight, finding that Aβ_42_ but not Aβ_16_ treatment induced significant neurite degeneration as well as the death of more than 60% of neurons, with degenerative changes being observed after 5 h of treatment (Figure 4A).

We then asked whether potassium efflux was required for Aβ-induced toxicity by increasing the extracellular potassium concentration in the culture medium from the normal 5–25 mM. First, we recorded from cultured hippocampal neurons and found that raising the level of extracellular potassium shifted the reversal potential of the GIRK current from −93 ± 4 to −36 ± 2.2 mV (*n* = 4). With the same change in potassium, the resting membrane potential depolarized from −81 ± 4 to −40 ± 2 mV. Importantly, the resting membrane potential shifted from being more positive than the equilibrium potential for potassium to being close to this equilibrium potential. Using the measured current-voltage relationships, the expected GIRK current at the resting membrane potential in low potassium was +21.2 ± 10 pA whereas in high potassium it was −9.6 ± 8.9 pA, i.e., there was virtually no outward current. We then raised the extracellular potassium concentration in the culture medium and found that it inhibited Aβ-induced death of neurons (Figure 4B). Similarly, blocking GIRK channels with the GIRK channel inhibitor tertiapin which significantly inhibited the Aβ_42_-induced potassium efflux (Figure 3B), also inhibited neuronal death (Figures 4A,C).

**Figure 5:**
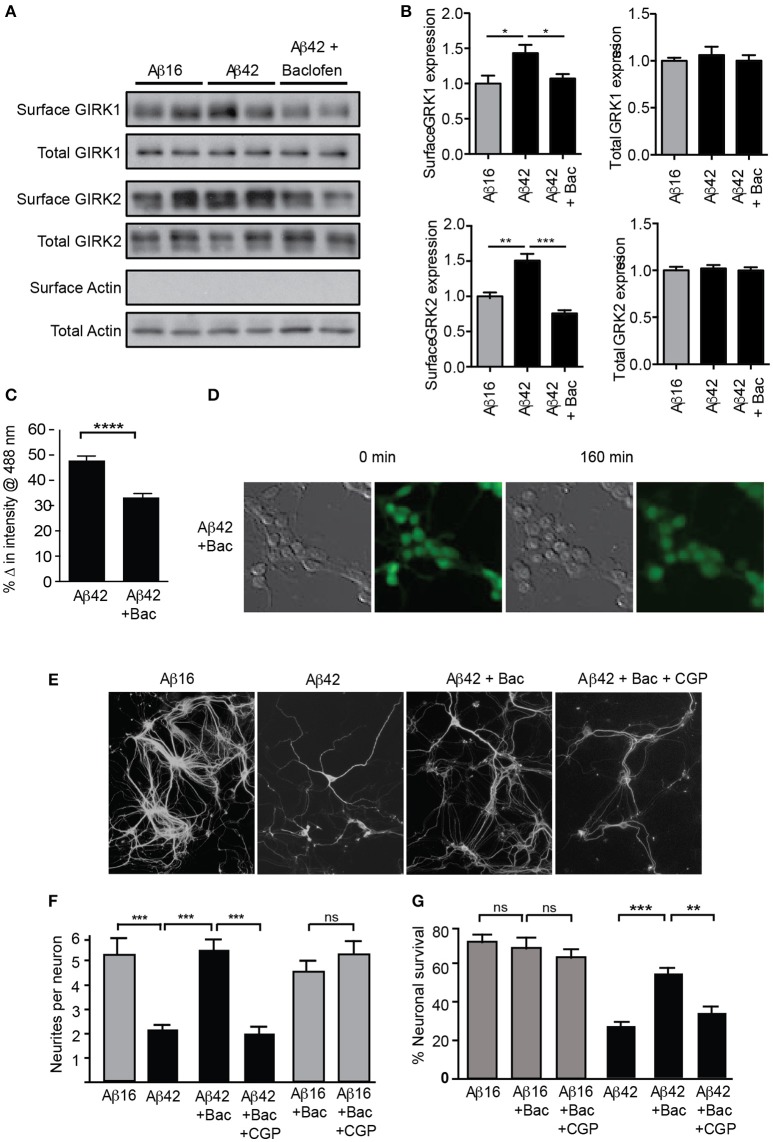
GIRK channel down-regulation inhibits Aβ_42_-induced neuronal degeneration. Western blots **(A)** and quantification **(B)** of total and surface GIRK1 and GIRK2 protein levels in mature hippocampal neurons treated for 2 h with either control Aβ_16_ or oligomeric Aβ_42_ and baclofen (*N* = 4 experiments). Baclofen treatment reversed the Aβ-induced upregulation of surface GIRK channel subunits. **(C)** Potassium loss from cultured neurons treated with Aβ_42_ was reduced by co-treatment with baclofen (*n* = 443 neurons). **(D)** Representative relief contrast and fluorescence (Asante Potassium Green-2) photographs of neuronal cultures taken immediately and 160 min after Aβ and baclofen treatment. **(E)** Photomicrographs of hippocampal cultures immunostained for β-III tubulin 20 h after treatment with Aβ peptides, baclofen (Bac) and/or the GABA_B_ receptor antagonist CGP55845 (CPGt). **(F)** Quantification of neurite integrity of the treated cultures (*N* = 3 experiments). **(G)** Percentage survival of neurons cultured in the presence of Aβ and baclofen for 20 h. Down-regulation of GIRK channels by chronic baclofen treatment inhibited cell death, but the neurotoxicity of Aβ_42_ was restored when neurons were co-cultured with the GABA_B_ receptor antagonist CGP55845 (CGP; *N* = 5 experiments). ^*^p < 0.05, ^**^p < 0.01, ^***^p < 0.001, ^****^p < 0.0001, *ns*, not significant.

We therefore asked whether increased surface expression and activity of GIRK channels was a requirement for this Aβ-induced neurotoxicity. We reasoned that sustained stimulation of GABA_B_ receptors, which are tightly coupled to GIRK channels, may lead to channel desensitization, and/or endocytosis and degradation of the entire receptor-channel complex, such as occurs for similar receptor-channel complexes (Clancy et al., 2007; Fowler et al., 2007; Raveh et al., 2010). Consistent with this idea, we found that a chronic 2 h treatment of cultured neurons with the GABA_B_ agonist baclofen reversed the upregulation of GIRK1 and 2 subunits on the surface of neurons induced by Aβ treatment, without changing the total GIRK subunit level (Figures 5A,B). Chronic baclofen treatment also inhibited the loss of potassium from Aβ-treated cells (Figures 5C,D). Furthermore, baclofen treatment significantly inhibited neuronal degeneration in long-term cultures (Figures 5E,F) and neuronal death (Figure 5G) induced by Aβ_42_ over 24 h in short-term cultures.

Taken together, these results indicate that potassium efflux through GIRK channels is a major mediator of Aβ_42_-induced neurotoxicity in hippocampal cultures and suggests that Aβ induces the upregulation of GIRK channel activity, thereby resulting in potassium efflux, reduced intracellular potassium, and apoptosis.

### Death signaling is mediated by p75^NTR^ signals

Despite an increase in GIRK channels at the cell surface, their typical activation by neurotransmitter-mediated Gβγ (e.g., following activation of GABA_B_ receptors) does not lead to a sustained potassium efflux or the lowering of intracellular potassium levels sufficient to promote cell death (see Section Discussion). However, we have previously demonstrated that p75^NTR^ can cause pathological activation of GIRK channels via upregulation of PIP_2_ levels (Coulson et al., 2008), which does not require Gβγ and which is necessary and sufficient for GIRK channel activation (Zhang et al., 1997; Huang et al., 1998). Furthermore, several groups, including ours, have reported a key role for p75^NTR^ in Aβ-induced neuronal degeneration (Sotthibundhu et al., 2008; Yang et al., 2008).

**Figure 6:**
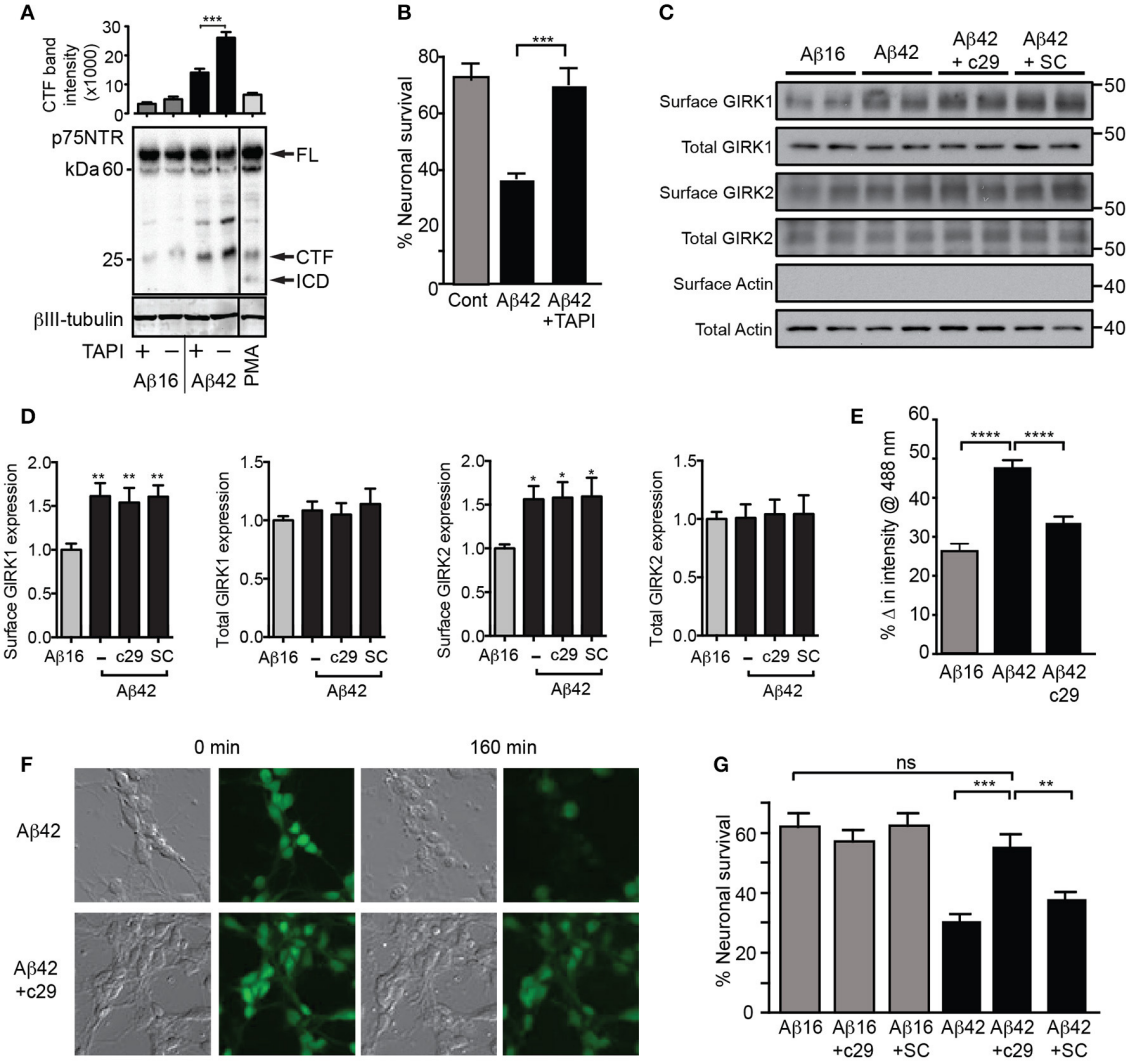
Aβ_42_-induced potassium efflux and apoptosis are mediated by p75^NTR^. **(A)** Western blot of p75^NTR^ cleavage in the presence of Aβ and TAPI or the cleavage stimulator PMA (positive control) for 3 h and quantification of the C-terminal fragment (CTF) band intensity (FL, full length; ICD, intracellular domain fragment; *N* = 2 experiments). **(B)** Percentage survival of neurons cultured in the presence of Aβ and treated with the metalloprotease inhibitor TAPI for 20 h, which significantly inhibited Aβ-induced cell death. Western blots **(C)** and quantification **(D)** of total and surface GIRK1 and GIRK2 protein levels in hippocampal neurons treated for 2 h with either control Aβ_16_ or oligomeric Aβ_42_and c29 or scrambled (SC) peptides (*N* = 8 replicates). Neither peptide treatment altered the levels of Aβ-induced upregulation of surface GIRK channel subunits. **(E)** Average decrease in potassium concentration of individual neurons in cultures treated for 160 min with Aβ_42_ and the dominant-negative p75^NTR^ peptide c29 (*n* = 458 neurons). **(F)** Representative relief contrast and fluorescence (Asante Potassium Green-2) photographs of neuronal cultures taken immediately and 160 min after Aβ and c29 treatment. **(G)** Percentage survival of neurons cultured in the presence of Aβ and control or c29 peptides over 20 h. c29 but not a scrambled peptide inhibited Aβ_42_-initiated death (*N* = 3 experiments). ^*^p < 0.05, ^**^p < 0.01, ^***^p < 0.001, ^****^p < 0.0001, *ns*, not significant.

As p75^NTR^-mediated PIP_2_ generation and GIRK channel-activating signals were previously found to be dependent on metalloprotease cleavage of p75^NTR^ to its C-terminal fragment (Coulson et al., 2008), we determined whether Aβ_42_ treatment induced p75^NTR^ proteolysis. Aβ_42_ but not Aβ_16_ stimulated increased generation of the p75^NTR^ C-terminal fragment (Figure 6A). Furthermore, an inhibitor of the p75^NTR^ metalloprotease (TAPI) blocked both Aβ-induced cleavage of p75^NTR^ and neuronal death (Figures 6A,B), indicating that activation of p75^NTR^ is necessary for Aβ-induced cell death.

Finally, we treated neurons with a cell-permeable peptide inhibitor of the p75^NTR^ C-terminal fragment that initiates the GIRK channel activity pathway (Coulson et al., 2008) and which can act by a dominant-negative mechanism to prevent death signaling (Coulson et al., 2000). Treatment of neurons with this peptide, c29, had no effect on Aβ-induced cell surface expression of GIRK subunits (Figures 6C,D), but significantly inhibited the enhanced potassium efflux from neurons co-treated with Aβ_42_ (Figures 6E,F). Furthermore, c29 peptide treatment significantly inhibited Aβ_42_-induced neuronal death, whereas a cell-permeable scrambled control peptide had no effect (Figure 6G). Together, these results suggest that p75^NTR^-mediated signals induced by Aβ_42_are concomitantly required for potassium efflux through GIRK channels and subsequent neuronal death.

## Discussion

Here we describe a novel, Aβ-triggered apoptotic pathway in which exposure to excitotoxic Aβ leads to upregulation and activation of GIRK channels, causing sustained potassium efflux from neurons and resulting in their apoptosis. Although, upregulation of GIRK channels is required for this death signaling, it appeared that this is not sufficient, with coincident activation of p75^NTR^ signaling by Aβ also being necessary for neuronal degeneration.

### Surface GIRK channels are upregulated by Aβ

Our first finding is that Aβ mediates the upregulation of GIRK channel expression on the neuronal plasma membrane. Although, the reason for this was not investigated, it has previously been observed that robust activation of NMDA receptors leads to an increase in cell surface recruitment of GIRK channels (Chung et al., 2009a,b). Analogous to this situation, Aβ can raise intracellular calcium levels by an NMDA receptor-dependent mechanism, and Aβ has also been shown to cause aberrant NMDA receptor activity and hyperexcitability (Palop et al., 2007). Consistent with this, the NMDA receptor antagonist APV was observed to block Aβ-induced calcium influx, as well as GIRK-mediated potassium efflux. It is therefore possible that GIRK channels are upregulated by such mechanisms in the current study (Chung et al., 2009b; Yao et al., 2013).

An acute effect of the upregulation of GIRK channels was enhanced slow inhibitory neurotransmission within the hippocampal CA1 circuit. Recruitment of GIRK channels to the plasma membrane is also a critical step in the depotentiation of NMDA-receptor-driven LTP in the hippocampus (Chung et al., 2009a,b). Therefore, Aβ-mediated upregulation of GIRK channels likely causes persistent depression of synaptic activity as observed in the mouse model of Down's syndrome (Ts65Dn). GIRK currents recorded in hippocampal neurons of these animals, which overexpress GIRK channels due to an additional copy of the GIRK2 gene (in addition to the amyloid precursor protein gene), are significantly more sensitive to inhibitory input through GABA_B_ receptors, which others have shown leads to an impairment of excitatory input and cognitive deficits (Harashima et al., 2006; Best et al., 2007). Moreover, a recent report demonstrated that Aβ perfusion of hippocampal slices caused increased resistance of CA3 neurons to firing, an effect which was mediated by GABA_B_ and GIRK channel activity (Nava-Mesa et al., 2013). Our findings provide an explanation for previous reports that, although Aβ is excitotoxic, neurons exposed to Aβ become synaptically silent prior to their death (Palop et al., 2007; Palop and Mucke, 2010; Yao et al., 2013).

### Upregulated GIRK channel activity is necessary for Aβ-induced cell death

The upregulation of GIRK channels by Aβ resulted in significant potassium efflux, demonstrated using electrophysiology and potassium imaging, that was required for subsequent neuronal death. In addition, blocking GIRK channels with tertiapin, or potassium efflux more generically, was sufficient to prevent this Aβ-induced potassium loss and subsequent neuronal degeneration. Furthermore, chronic baclofen treatment also blocked Aβ-induced potassium efflux, prevented neuronal degeneration, and down-regulated surface GIRK channel expression. Although, it is possible that the baclofen treatment masked the toxic effect of Aβ by an unrelated mechanism, taken together with our other data, the correlation between baclofen-induced down-regulation of GIRK channel activity and reduced Aβ-toxicity is consistent with a causal relationship. Potassium dysregulation is a feature of Alzheimer's disease brain tissue (Roberts et al., 2016), and similar to our findings, others have shown that potassium efflux from cortical neurons can be promoted by Aβ, and that this efflux is required to cause subsequent degeneration, although the mechanism remains unclear (Yu et al., 1998; Shabala et al., 2010). Our data indicate that Aβ_42_ treatment causes sufficient potassium to leave the cell to reduce the intracellular potassium concentration to approximately half (80 mM), which in turn is permissive for the apoptosome to assemble and caspases to be activated, a finding that is consistent with previous reports (Cain et al., 2001; Coulson et al., 2008). In addition, our results strongly indicate that GIRK channels are key mediators of this critical apoptotic step. However, our findings do not rule out the possible involvement of other potassium channels in Aβ-mediated neurotoxicity.

### Coincident activation of p75^NTR^ and GIRK channels is required to induce cell death

Although, upregulation of membrane GIRK channels and increased potassium efflux were directly associated with Aβ-induced neuronal degeneration, it remains unclear whether the pathological GIRK channel activation occurred by inhibitory neurotransmission and/or through a coincident Aβ activated pathway—such as that mediated by p75^NTR^. Although, neurotransmitter receptor activation may play a contributory role in the observed potassium efflux, chronic stimulation of GABA_B_ receptors inhibited cell death, coincident with GIRK channels being removed from the cell surface. Likewise, it is unlikely that the channels were activated by G_q_-coupled neurotransmitter receptor-mediated mechanisms, as these cause the hydrolysis of PIP_2_ and inhibit channel activity (Raveh et al., 2010), with PIP_2_ being required for GIRK channel opening. Several groups have previously reported that p75^NTR^ can increase PIP_2_ levels via Rac1 (Gibon et al., 2015; Zeinieh et al., 2015), a necessary step for GIRK channel activation by p75^NTR^ (Coulson et al., 2008). We therefore suggest that p75^NTR^ mediates pathological GIRK channel activity subsequent to Aβ-induced channel upregulation, nominally ~2–3 h after Aβ application.

Regardless of whether or not p75^NTR^ is directly responsible for GIRK channel activity, p75^NTR^ signaling is required for Aβ-induced cell death, as blocking this signaling, even in the context of enhanced surface GIRK expression, prevented both loss of cellular potassium and cell death. Aβ has been widely reported to activate p75^NTR^ either directly or indirectly, leading to neuronal degeneration; neurons with reduced p75^NTR^ expression or function are resistant to Aβ-induced toxicity *in vitro* and *in vivo* (Yaar et al., 1997; Ivins et al., 1998; Tsukamoto et al., 2003; Sotthibundhu et al., 2008; Yang et al., 2008; Knowles et al., 2009; Yu et al., 2012). However, because p75^NTR^ can regulate a range of signaling pathways, the mechanism by which it mediates apoptosis in response to Aβ has remained unclear (Coulson, 2006; Skeldal et al., 2011).

**Figure 7:**
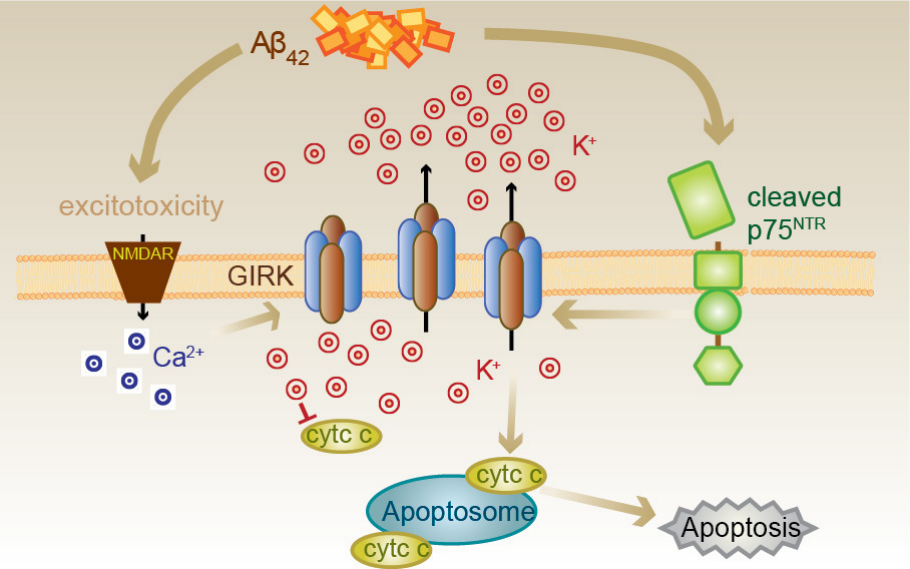
Proposed Aβ_42_-p75^NTR^-GIRK channel death signaling pathway. Aβ_42_ excitotoxicity increases intracellular calcium in neurons via NMDA receptors (NMDAR), which triggers an increase in the number of GIRK channels at the cell surface. Aβ_42_ also results in the cleavage of p75^NTR^ to its C-terminal fragment, which can then activate the GIRK channels resident on the surface to promote a pathological potassium efflux. Lowered internal potassium concentration removes inhibition of apoptosome formation, thereby causing cell death. Cell death can be inhibited by raised extracellular potassium, the GIRK channel inhibitor tertiapin, chronic baclofen treatment removing GIRK channels from the cell surface, preventing p75^NTR^ cleavage or treatment with a p75^NTR^ signaling inhibitor (c29).

We suggest that Aβ causes the induction of GIRK channel surface expression which, when activated acutely by traditional G-protein-coupled receptors, enhances inhibitory neurotransmission (Figure 7). However, sustained exposure to Aβ coincidently results in activation of p75^NTR^ cleavage and signaling pathways, one of which involves the activation of GIRK channels. As activation of GIRK channels by p75^NTR^ occurs through increased levels of PIP_2_ and independently of G-proteins (Coulson et al., 2008), potassium efflux through GIRK channels could be sustained (Raveh et al., 2010). Sustained channel activity resulting in substantial potassium efflux then triggers apoptosis. However, alternatively, or coincident with p75^NTR^-mediated GIRK channel activity, other p75^NTR^-mediated signals (e.g., activation of c-jun kinase; Coulson, 2006) could facilitate the activation of the apoptosome via the mitochondrial death pathway in the context of already lowered intracellular potassium mediated via GIRK channels by other means.

### Summary

In conclusion, we have demonstrated that exposure to excitotoxic Aβ drives an increase in the number of surface GIRK channels which, when activated, can directly result in increased inhibitory neurotransmission and neuronal circuit silencing. Over a longer timescale, GIRK channel activity is a key mediator of neuronal degeneration and apoptosis. However, for this novel cell death pathway to proceed, it also requires signaling by p75^NTR^, which can be coincidently triggered by Aβ exposure, resulting in pathological GIRK channel activity.

## Ethics statement

All animal procedures were approved by the University of Queensland Animal Ethics Committee in accordance with the Australian code for the care and use of animals for scientific purposes (2013).

## Author contributions

LM, VA, HG, PS, and EC designed the experiments and wrote the manuscript; LM, HG, VA, DM, SJ, and GK performed experiments; LM, HG, VA, DM, FM, and PS analyzed data and all authors discussed the work and commented on the manuscript.

### Conflict of interest statement

The authors declare that the research was conducted in the absence of any commercial or financial relationships that could be construed as a potential conflict of interest.
